# Care of the Cervix after Labor

**Published:** 1915-12

**Authors:** 


### Care of the Cervix After Labor.

This article embodies the plea of a victim of carcinoma of the cervix for her sisters. It ought to be eloquent with her sincerity and lack of bitterness at her own fate, but no attempt will be made to make it so by mere words, while the writer is unable to inject into it anything of scientific or technical value. It is a dictum of gynaecology that uterine cancer is a disease of parous, fibroids of nulliparous women of middle and elderly life. Like many another dictum, it states not an absolute fact but still a close enough approach to truth to have weight.

Social conditions have so changed within the last two generations that the woman of middle life, who has reared her family, is no longer merely a wife, mother and keeper of a home. With abundant experience, often increased means for leisure from routine tasks, with matured brain and body, she is just about to enter a field of wider usefulness to her family, now reaching to the third generation, to her acquaintances, and by her interest in educational, sociologic and even business and, to some degree, political activities, to the state. The loss of a child, of a young and inexperienced woman, while causing equal grief and regret, is from the standpoint of society, potential rather than actual. Economic conditions have so altered that, on the one hand, the loss of a man no longer necessarily involves the financial break-down of a family while, for the same reason, the loss of a woman has increased in its economic importance. The present problems of society include, in a greater degree than ever before, individual and educational effort toward economics of the home; moral prophylaxis; enforcement of details of sanitation and hygiene; guidance of the dependent classes in various ways. For all of these problems, without considering at all the possible engagement of women in politics, society is very largely dependent upon women. Even if taking no active vocational part in the various movements bearing on these problems, if nominally merely the housewife, dependent on her husband for support, the average, intelligent, retiring woman of the home still has leisure, tact and practical knowledge for aiding in carrying out the necessary reforms, far more than the average, equally intelligent husband who is engrossed in his business. In fact, there has been no time in the world's history when progress along right lines has depended so largely on normal womanhood as it does at present and will in the near future.

Without underestimating the importance of venereal diseases, especially of married women, without advocating the suspension of child-bearing or ignoring the fact that, in every sense, physical, mental and social, woman should be regarded broadly and not in the narrow sexual sense, the fact remains that procreation involves a higher tax than it should. Pregnancy is not a disease but it borders closely on the pathologic. It still has a direct mortality of at least one per cent—more than some infections with bacteria—and its indirect mortality is probably not far from ten per cent in the aggregate for the period of active marital life. In other words, unless too high a rate has been set, the several pregnancies of the average woman involve about the same risk as an attack of typhoid.

Prompt attention to the perineum has greatly reduced invalidism and, thus indirectly, mortality, and it has increased the usefulness of women and the general economic welfare of the family. Without overlooking the fact that cervical laceration is even less preventable than that of the perineum, nor disregarding the inevitable difficulties of diagnosis and the dangers of routine interference from drawing too close an analogy betwen the two traumatisms, we cannot refrain from suggesting that the subject of cervical laceration is a vital issue, sociologic as well as surgical, and that its importance should not be hidden by too firm a belief in the availability of late methods of cure of existing and prophylaxis of threatened lesions.

